# Longitudinal Outbreak of Multidrug-Resistant Tuberculosis in a Hospital Setting, Serbia

**DOI:** 10.3201/eid2503.181220

**Published:** 2019-03

**Authors:** Irena Arandjelović, Matthias Merker, Elvira Richter, Thomas A. Kohl, Branislava Savić, Ivan Soldatović, Thierry Wirth, Dragana Vuković, Stefan Niemann

**Affiliations:** University of Belgrade, Belgrade, Serbia (I. Arandjelović, B. Savić, I. Soldatović, D. Vuković);; Leibniz-Zentrum für Medizin und Biowissenschaften, Borstel, Germany (M. Merker, T.A. Kohl, S. Niemann);; Laboratory Limbach, Heidelberg, Germany (E. Richter); Paris Sciences & Lettres University, Paris, France (T. Wirth);; Sorbonne Universités, Paris (T. Wirth); German Center for Infection Research, Borstel, Germany (S. Niemann)

**Keywords:** tuberculosis, multidrug-resistant, MDR TB, whole-genome sequencing, outbreak, transmission, antimicrobial resistance, Serbia, tuberculosis and other mycobacteria, bacteria

## Abstract

A retrospective population-based molecular epidemiologic study of multidrug-resistant *Mycobacterium tuberculosis* complex strains in Serbia (2008–2014) revealed an outbreak of TUR genotype strains in a psychiatric hospital starting around 1990. Drug unavailability, poor infection control, and schizophrenia likely fueled acquisition of additional resistance and bacterial fitness–related mutations over 2 decades.

The overall burden of tuberculosis (TB) in Serbia has been greatly reduced in recent years (*1**,**2*). However, a recent study revealed transmission of multidrug-resistant (MDR) *Mycobacterium tuberculosis* complex (MTBC) strains (i.e., MTBC strains resistant to at least rifampin and isoniazid) in Belgrade (*3*). In addition, data retrieved from the national database of MDR TB patients indicate a concentrated burden of MDR TB and extensively drug-resistant (XDR) TB, defined as additional resistance to 1 fluoroquinolone and 1 of the 3 injectable second-line drugs, among psychiatric inpatients in Serbia. To gain more insights into countrywide transmission routes, strain dynamics, and bacterial evolution over time, we retrospectively investigated all (n = 110) patients who received a diagnosis of MDR TB during January 1, 2008–May 31, 2014, in Serbia. 

## The Study

We subjected 1 MTBC isolate per patient to phenotypic drug susceptibility testing and whole-genome sequencing (WGS) (Appendix 1). We retrieved patients’ demographic, epidemiologic, and clinical data from the national database of MDR TB patients, as well as from their medical and laboratory records.

Most patients were male (87/110, 79.1%) and born in Serbia (107/110, 97.3%); mean age was 49.5 years (range 15–83). We observed concurrent conditions for 55 patients; schizophrenia was the most prevalent (26/55, 47.3%). Of the 110 patients, 61 (55.5%) had previously experienced TB. Susceptibility testing results showed that 19/110 (17.3%) MDR MTBC isolates were resistant to all first-line drugs, and 11/110 (10.0%) were classified as XDR (Appendix 1 Table 1). We successfully completed WGS for 103/110 isolates, representing 93.6% of all MDR TB cases recorded over the study period. 

**Figure 1:**
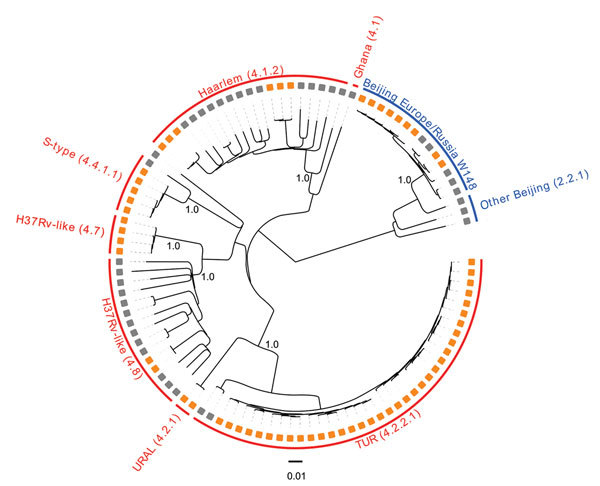
Maximum-likelihood phylogeny, applying a general time-reversible substitution model, of 103 multidrug-resistant (MDR) *Mycobacterium tuberculosis* complex (MTBC) isolates from Serbia sampled during 2008–2014. Orange squares indicate MDR MTBC isolates associated with putative transmission chains (molecular clusters); gray squares indicate other MDR MTBC isolates. All analyzed strains belong to the major MTBC phylogenetic lineage 4 (Euro-American) or lineage 2 (Beijing); red text indicates lineage 4 and blue text, lineage 2. Subgroups are further named according to the single-nucleotide polymorphism barcode nomenclature from Coll et al. (*5*), and to the associated mycobacterial interspersed repetitive unit–variable-number tandem-repeat genotype classification (*6*). Subgroup-defining branches are labeled with bootstrap values based on 1,000 resamples.

We considered 6,512 single-nucleotide polymorphisms (SNPs) differentiating all isolates to analyze their phylogenetic relationships. The MDR MTBC strain population comprised 37/103 (35.9%) isolates classified as lineage 4.2.2.1 (TUR genotype), 20/103 (19.4%) isolates of lineage 4.1.2 (Haarlem genotype), 17/103 (16.5%) isolates of lineage 2.2.1 (Beijing genotype), 15/103 (14.6%) isolates of lineage 4.8 (H37Rv-like strains), 8/103 (7.8%) isolates of lineage 4.4.1.1 (S-type), 2/103 (1.9%) isolates of lineage 4.2.1 (URAL genotype), 1 isolate of lineage 4.1 (Ghana), and 1 nonclassified lineage 4 isolate. Among lineage 2.2.1 Beijing isolates, the previously described Europe/Russia W148 MDR outbreak isolates (*4*) were most prevalent, present in 14/17 (82.4%) of the cases (Figure 1; Appendix 2).

Seeking to identify recent chains of transmission, we defined molecular clusters as surrogate markers for epidemiologically linked cases (Appendix 1). Overall, 63/103 (61.2%) isolates could be assigned to 12 different clusters, each including 2–17 patients. The 2 largest clusters, 1 containing 14 and 1 containing 17 cases, comprised isolates of TUR genotype; the next-largest cluster was of 7 Beijing Europe/Russia W148 isolates. For all 63 suggested epidemiologic links, we were able to retrospectively identify 40 (63.5%) epidemiologic links (e.g., household and social contacts) (Appendix 1 Figure 1).

**Figure 2:**
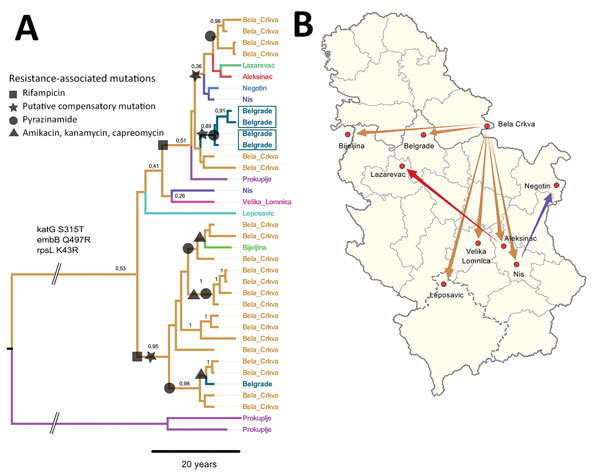
Most likely temporal and spatial origin of *Mycobacterium tuberculosis* complex (MTBC) TUR genotype outbreak strains in Serbia. A) Location annotated time-scaled phylogeny (maximum clade credibility tree) derived from a Bayesian discrete trait phylogeographical analysis of 37 lineage 4.2.2.1 (TUR genotype) multidrug-resistant (MDR) MTBC isolates. Branches are color-coded according to the most likely place of infection, assuming a fast-progression hypothesis (Appendix 1). Branches are annotated with location probabilities; symbols represent acquisition of individual resistance-related mutations shared by all derived strains. B) Regional and countrywide spread of individual TUR genotype outbreak strains originating from Bela Crkva Hospital. Arrows indicate inferred location changes determined from the genealogy shown in panel A. Scale bar indicates nucleotide substitutions per site.

Our main finding was that 35/37 (94.6%) TUR isolates shared identical mutations that confer drug resistance to isoniazid (*katG* S315T), streptomycin (*rpsL* K43R), and ethambutol (*embB* Q497R); we therefore classified them as TUR-outbreak isolates. TUR-outbreak isolates further differentiated into 2 individual transmission chains characterized by 2 distinct rifampin resistance–mediating mutations: *rpoB* S450W in 1998 (95% highest posterior density [HPD] 1993–2001) and *rpoB* S450L in 2003 (95% HPD 2000–2005) (Figure 2, panel A; Appendix 1 Figure 2). Subsequently, both strain populations acquired individual mutations in other RNA polymerase genes (*rpoA* P25R, *rpoC* V431M, and *rpoC* F452L), which have been proposed to enhance the in vitro growth rate of rifampin-resistant strains (*7*). Furthermore, *rpoA* mutations in the entire dataset were more likely to arise in clustered isolates than in unique isolates (20/63 vs. 1/40; p<0.001), thus indicating their ability to restore fitness of *rpoB* mutants, increase transmission success, or both.

Of the 35 TUR-outbreak isolates, 26 (74.3%) were from patients hospitalized in Bela Crkva (BC) Hospital, the national center for treatment of all psychiatric patients with concomitant respiratory illnesses. Of note, 22 (84.6%) of these 26 patients had been transferred from 7 different psychiatric hospitals to BC Hospital for pulmonary diagnosis and treatment; 5 were admitted at BC Hospital with either confirmed or suspected TB diagnosis (Appendix 2). Screening for TB at time of admission had not been implemented in BC Hospital during the study period. 

To determine the geographic origin of the 3-fold resistant TUR ancestor and to test for the putative independent introduction of 2 different rifampin-resistant cases to the BC Hospital from other hospitals, we extended our Bayesian approach with a discrete trait model introducing the likely place of infection for each patient. We used 2 assumptions: first, a fast disease progression assumed infection and diagnosis of MDR TB within the first 2 years after admission to BC Hospital; and second, a slow disease progression in which patients who received a diagnosis within 2 years after admission were identified as latent MDR TB cases, meaning they had contracted the infection in their hometown or a previous hospital.

The comparison of both models using path sampling clearly favored the fast progression model, suggesting the origin of the TUR outbreak in BC Hospital with a probability of 53% (i.e., node location probability; second likely origin was Belgrade, 12%) (Figure 2, panel A). The 2 unique rifampin-resistance mediating mutations were also more likely to have originated in BC Hospital itself (51% for *rpoB* S540W node, 95% for *rpoB* S540L node, and <15% for other location probabilities). Individual transmission events occurred to remote cities but also within Belgrade (Figure 2, panels A, B). In comparison, applying the slow TB progression hypothesis, TUR outbreak strains would have been imported multiple times from different regions throughout the country to BC Hospital, with node location probabilities <10% for all locations (Appendix 1 Figure 3). Tracing the time of hospitalization at BC Hospital and MDR TB diagnosis of patients infected with TUR strains backward revealed that the 2 clades (defined by *rpoB* S450L and *rpoB* S450W) indeed coexisted over 2 decades (Appendix 1 Figure 4).

## Conclusions

In a retrospective approach using WGS-based molecular epidemiology, Bayesian statistics, and detailed epidemiologic investigations, we show that MDR TB in Serbia is associated with nosocomial transmission at BC Hospital, likely accompanied by a fast progression to disease within 2 years. Drug unavailability in the 1990s (*8*), schizophrenia as a recognized cause of unsuccessful completion of TB treatment (*9*), and long-term and repeated hospitalizations under extremely adverse living conditions (*10*), together with the absence of a TB infection control program, are believed to be the main drivers of the evolutionary trajectories and success of TUR-outbreak strains in Serbia. The TUR-outbreak strain was considered intrinsically resistant to 3 first-line drugs and probably acquired an MDR genotype in 2 independent events in BC Hospital during the 1990s. Subsequently, putative compensatory mechanisms were selected, the strain acquired individual XDR genotypes, and it spread into other settings in Serbia by family contacts and other modes.

Detection of the extensive transmission network in BC Hospital led to the development and implementation of an appropriate TB infection control program featuring the use of rapid laboratory tests for prompt detection of new cases, completion of appropriate second-line treatment regimens, and markedly expanded contact tracing activities. Since 2015, only 1 new case of MDR TB has been recorded in BC Hospital. However, MDR TB transmission in the general population must continue to be carefully monitored.

Appendix 1 Additional methods and information about a longitudinal study of MDR TB in a hospital setting, Serbia.

Appendix 2 Additional data for longitudinal study of MDR TB in a hospital setting, Serbia.

## References

[1] World Health Organization. Global tuberculosis report. Contract no.: WHO/HTM/TB/2017.23. Geneva: The Organization; 2017.

[2] European Centre for Disease Prevention and Control; WHO Regional Office for Europe. Tuberculosis surveillance and monitoring in Europe 2017. Stockholm: European Centre for Disease Prevention and Control; 2017.

[3] Vuković D, Rüsch-Gerdes S, Savić B, Niemann S. Molecular epidemiology of pulmonary tuberculosis in belgrade, central serbia. J Clin Microbiol. 2003;41:4372–7. 10.1128/JCM.41.9.4372-4377.2003 12958271 PMC193815

[4] Merker M, Blin C, Mona S, Duforet-Frebourg N, Lecher S, Willery E, et al. Evolutionary history and global spread of the *Mycobacterium tuberculosis* Beijing lineage. Nat Genet. 2015;47:242–9. 10.1038/ng.3195 25599400 PMC11044984

[5] Coll F, McNerney R, Guerra-Assunção JA, Glynn JR, Perdigão J, Viveiros M, et al. A robust SNP barcode for typing *Mycobacterium tuberculosis* complex strains. Nat Commun. 2014;5:4812. 10.1038/ncomms5812 25176035 PMC4166679

[6] Niemann S, Merker M, Kohl T, Supply P. Impact of genetic diversity on the biology of *Mycobacterium tuberculosis* complex strains. Microbiol Spectr. 2016;4:TBTB2-0022-2016. 10.1128/microbiolspec.TBTB2-0022-2016 27837742

[7] Comas I, Borrell S, Roetzer A, Rose G, Malla B, Kato-Maeda M, et al. Whole-genome sequencing of rifampicin-resistant *Mycobacterium tuberculosis* strains identifies compensatory mutations in RNA polymerase genes. Nat Genet. 2011;44:106–10. 10.1038/ng.1038 22179134 PMC3246538

[8] Jovanovic D, Skodric-Trifunovic V, Markovic-Denic L, Stevic R, Vlajinac H. Clinical and epidemiological evaluation of tuberculosis in Serbia, 1990–2004. Int J Tuberc Lung Dis. 2007;11:647–51.https://www.ncbi.nlm.nih.gov/entrez/query.fcgi?cmd=Retrieve&db=PubMed&list_uids=17519096&dopt=Abstract 17519096

[9] Pachi A, Bratis D, Moussas G, Tselebis A. Psychiatric morbidity and other factors affecting treatment adherence in pulmonary tuberculosis patients. Tuberc Res Treat. 2013;2013:489865. 10.1155/2013/489865 PMC3649695 23691305

[10] Tosevski DL, Gajic SD, Milovancevic MP. Mental healthcare in Serbia. Int Psychiatry. 2010;7:13–5. 10.1192/S1749367600000941 31508020 PMC6734953

