# Supplementary material for: Longitudinal Outbreak of Multidrug-Resistant Tuberculosis in a Hospital Setting, Serbia
Source: Emerg Infect Dis. 2019 Mar;25(3):555–8. doi: 10.3201/eid2503.181220 (PMC6390760; doi:10.3201/eid2503.181220)
Supplement: Appendix 1 — Additional methods and information about a longitudinal study of MDR TB in a hospital setting, Serbia. [file 18-1220-Techapp-s1.pdf]

Article DOI: https://doi.org/10.3201/eid2503.181220

Longitudinal Outbreak of Multidrug-Resistant Tuberculosis in a Hospital Setting, Serbia

Appendix 1

Materials and Methods

Bacterial Strains and Drug Susceptibility Testing (DST)

One isolate per patient was included in the study. The isolates were stored in glycerol stocks at −70°C, and re-cultured on Löwenstein-Jensen (LJ) media for 4–6 weeks at 37°C before further testing. Identification of the isolates was verified by the GenoType MTBC assay (Hain Lifescience, https://www.hain-lifescience.de/en/). DST for first- (except pyrazinamide (PZA)) and second-line drugs was performed with the indirect proportion method on LJ slants using World Health Organization (WHO) recommended critical concentrations during 2008–2011, and as described previously (*1*). PZA was tested in MGIT960 according to the manufacturer instructions and at WHO recommended critical concentrations. From 2012 onward, all drugs, except cycloserine (CS) and para-aminosalicylic acid (PAS), were tested in MGIT960. Both CS and PAS were still tested on LJ slants at 30 mg/L, and 1 mg/L with the indirect proportion method as recommended that time.

DNA Extraction

DNA extraction was performed by using QIAamp DNA Mini Kit (QIAGEN, http://www.qiagen.com) according to the manufacturer's instructions. Briefly, one loop of bacterial colonies grown on LJ medium was suspended in 0.2 mL of the tissue lysis buffer with vortexing. Suspension was centrifuged for 10 min at 7,500 rpm. Bacterial pellet was resuspended in 180 µL of the appropriate enzyme solution (20 mg/ml lysozime; 20 mM Tris-HCl, pH 8.0; 2 mM EDTA; 1.2% Triton), and incubated for 3h at 37°C. Twenty µLof Proteinase K and 200 µL of lysis buffer were added in each tube with vortexing, and all tubes were incubated at 56°C for

30 min, and then for a further 15 min at 95°C. After centrifugation, DNA extraction was completed following the manufacturer's tissue protocol (from step 4).

Whole-Genome Sequencing (WGS)

DNA library preparation was performed according to the Nextera XT manufacturer instructions (Illumina, http://www.illumina.com). Sequencing was carried out with the Illumina MiSeq and HiSeq system with a minimum average coverage depth of 50-fold. Fastq files have been submitted to the European Nucleotide Archive (ENA);accession numbers are given in Appendix 2. Resulting reads were mapped to the *M. tuberculosis* H37Rv genome (GenBank accession no. NC_000962.3) using BWA-MEM (*2*), and mappings refined with the GATK software package (*3*).

Variants were detected with Samtools (*4*) and filtered with perl scripts for thresholds of a minimum coverage of 4 reads in both forward and reverse orientation, 4 reads calling the allele with a phred score of ≥20, and 75% allele frequency. After exclusion of multiple consecutive variant calls (in a 12 bp window), variants in drug resistance associated genes or repetitive regions, the remaining positions that had a clear base call for all strains and matched the above mentioned threshold levels in at least 95% of all strains were considered as valid, and combined in a concatenated sequence alignment. Mutations in drug resistance associated genes were analyzed separately but considering the same thresholds, as mentioned above.

Maximum Likelihood (ML) Phylogenetic Reconstruction and Molecular Clusters

A ML tree was calculated, based on the concatenated sequence alignment using FastTree (*5*) with a general time reversible (GTR) substitution model and 1,000 resamples. A molecular cluster was defined as ≥2 strains within a maximum genome wide distance of 5 single nucleotide polymorphisms (SNPs). In case another strain exceeded this threshold but shared a common ancestor with other clustered isolates; we indicated this in Figure 1and in Appendix 2 but did not considered these few cases for the cluster rates.

Comparison of Bacterial Demographic and Molecular Clock Models

For a temporal calibration of phylogenetic trees, we used BEAST 2.4.2 (*6*) and a concatenated sequence alignment of 6,512 SNPs discriminating all isolates.

Prior to model comparisons within the Bayesian statistical framework, we sought for a proper substitution model using jmodeltest 2.1 (based on maximum-likelihood estimates) (*7*).

Akaike and Bayesian information criteria (AIC and BIC) were considered for the identification of appropriate substitution models (Appendix 1 Table 2).

To compare a strict versus a relaxed clock model (assuming a constant population size), and different demographic models under the best clock model, we ran each analysis with 300 million steps, sampled 5,000 trees, and discarded 10% as burn-in, resulting in ESS values for all parameters in the hundreds and thousands. Marginal likelihood estimates for each run were computed using path sampling (included in the BEAST 2.4.2 MODEL_SELECTION package) with an α of 0.3 and 50% burn-in with 10 million iterations resulting in ESS values in the hundreds. For model selection, we compared respective marginal likelihood estimates from 50 steps (Appendix 1 Table 3).

Discrete Phylogeographical Analysis

The spatial and temporal expansion of MTBC strains belonging to the TUR genotype, i.e., MTBC lineage 4.2.2, (classification inferred from Coll et al. (*8*), and Niemann et al. (*9*) was analyzed with BEAST 2.4.2 and the Geo_Sphere package,as described previously (*10*). For a discrete trait phylogeography analysis we used the likely place of infection for each patient to calculate location annotated time-scaled phylogenies. We used a coalescent constant size demographic model under a strict molecular clock fixed to 10^{-7} substitutions per site per year with a GTR substitution model without invariant sites and no gamma distribution, as well as a tip dating approach (best model for TUR genotype dataset, see below, Appendix 1 Table 3). A Chain length of 300 million ignoring a burn-in of 10%, 5,000 trees sampled, resulted in ESS values for all parameters in the thousands. The temporal and geographic distribution was visualized with SPREAD (*11*) using Google Earth for location mapping of internal nodes and inferred location changes on the basis of a maximum clade credibility (MCC) tree.

Rationales for Determination of Likely Place of Infection

To identify a possible source/origin for the identified MDR TUR outbreak, i.e., nosocomial transmission versus a possible introduction of a regional- or community-acquired MDR strain type, we compared 2 hypotheses (*1*). One was of a fast TB progression, assuming that all patients receiving an MDR TB diagnosis at the national center for treatment of all psychiatric patients with concomitant respiratory illnesses Bela Crkva (BC Hospital) have been infected in BC Hospital itself when there was >1 month between admission and diagnosis. If the

diagnosis was within the first month following admission at the BC Hospital, the initial hospital or the hometown of the patient was assumed as a likely place of infection (*2*). The second hypothesis we used was a slow TB progression hypothesis: when MDR TB was diagnosed within the first 2 years after the admission to BC Hospital, we assumed that a patient had been infected elsewhere and was transferred as a latent TB case. The diagnosis after 2 years was considered as a healthcare-associated infection in BC Hospital. The geographic inference of place of infection for both hypotheses was used in the discrete phylogeographic analysis, and marginal likelihoods, inferred by path sampling, were compared as described above.

Statistical Analysis

Categorical data were compared by Pearson's χ^2test, χ^2 test for trend, and Mann-Whitney U test (or Fisher exact test and Monte Carlo simulation when expected group sizes were less than five). All tests were performed as two-sided tests; p values <0.05 were considered statistically significant. We performed statistical analyses with SPSS version 20 (IBM, https://www.ibm.com/analytics/spss-statistics-software).

Ethics

The study protocol was reviewed and approved by the Ethical Committee, Faculty of Medicine, University of Belgrade, decision number 29/V-14.

DST Results

Phenotypic drug resistance rates, other than those for RIF and INH, among 110 MDR-MTBC isolates were as follows: 75.5% (83/110) were resistant to streptomycin (SM), 65.5% (72/110) to ethambutol (EMB), 51.8% (57/110) to pyrazinamide, 17.3% (19/110) to ofloxacin, 14.5% (16/110) to amikacin, and 15.5% (17/110) to capreomycin. Resistance to ethionamide was identified in 21/83 of the isolates (25.3%, 27 DST results were not available), 3/110 (2.7%) were tested resistant to para-aminosalicylic acid, while all isolates were susceptible to cycloserine.

References

1. Pardini M, Iona E, Varaine F, Karakozian H, Arzumanian H, Brunori L, et al.; LONG-DRUG Study Group. *Mycobacterium tuberculosis* drug resistance, Abkhazia. Emerg Infect Dis. 2005;11:501–3. PubMed http://dx.doi.org/10.3201/eid1103.040903

2. Li H, Durbin R. Fast and accurate short read alignment with Burrows–Wheeler transform. Bioinformatics. 2009;25:1754–60. PubMed http://dx.doi.org/10.1093/bioinformatics/btp324

3. McKenna A, Hanna M, Banks E, Sivachenko A, Cibulskis K, Kernytsky A, et al. The Genome Analysis Toolkit: a MapReduce framework for analyzing next-generation DNA sequencing data. Genome Res. 2010;20:1297–303. PubMed http://dx.doi.org/10.1101/gr.107524.110

4. Li H, Handsaker B, Wysoker A, Fennell T, Ruan J, Homer N, et al.; 1000 Genome Project Data Processing Subgroup. The Sequence Alignment/Map format and SAMtools. Bioinformatics. 2009;25:2078–9. PubMed http://dx.doi.org/10.1093/bioinformatics/btp352

5. Price MN, Dehal PS, Arkin AP. FastTree 2—approximately maximum-likelihood trees for large alignments. PLoS One. 2010;5:e9490. PubMed http://dx.doi.org/10.1371/journal.pone.0009490

6. Bouckaert R, Heled J, Kühnert D, Vaughan T, Wu CH, Xie D, et al. BEAST 2: a software platform for Bayesian evolutionary analysis. PLOS Comput Biol. 2014;10:e1003537. PubMed http://dx.doi.org/10.1371/journal.pcbi.1003537

7. Darriba D, Taboada GL, Doallo R, Posada D. jModelTest 2: more models, new heuristics and parallel computing. Nat Methods. 2012;9:772. PubMed http://dx.doi.org/10.1038/nmeth.2109

8. Coll F, McNerney R, Guerra-Assunção JA, Glynn JR, Perdigão J, Viveiros M, et al. A robust SNP barcode for typing *Mycobacterium tuberculosis* complex strains. Nat Commun. 2014;5:4812. PubMed http://dx.doi.org/10.1038/ncomms5812

9. Niemann S, Merker M, Kohl T, Supply P. Impact of genetic diversity on the biology of *Mycobacterium tuberculosis* complex strains. Microbiol Spectr. 2016;4:TBTB2–0022–2016.

10. Lemey P, Rambaut A, Drummond AJ, Suchard MA. Bayesian phylogeography finds its roots. PLOS Comput Biol. 2009;5:e1000520. PubMed http://dx.doi.org/10.1371/journal.pcbi.1000520

11. Bielejec F, Rambaut A, Suchard MA, Lemey P. SPREAD: spatial phylogenetic reconstruction of evolutionary dynamics. Bioinformatics. 2011;27:2910–2. PubMed http://dx.doi.org/10.1093/bioinformatics/btr481

Appendix 1 Table 1. Resistance profiles of 110 MDR MTBC strains in a longitudinal outbreak, Serbia*

Resistance to	Phenotypic resistance profile	No. isolates
2 drugs	INH, RIF	15
3 drugs	SM, INH, RIF	4
	INH, RIF, EMB	4
	INH, RIF, PZA	1
4 drugs	SM, INH, RIF, EMB	15
	SM, INH, RIF, PZA	5
	INH, RIF, EMB, PZA	4
	SM, INH, RIF, ETH	1
5 drugs	SM, INH, RIF, EMB, PZA	19
	SM, INH, RIF, PZA, ETH	10
	SM, INH, RIF, EMB, OFL	4
	SM, INH, RIF, EMB, PAS	1
	SM, INH, RIF, EMB, ETH	1
	INH, RIF, EMB, PZA, ETH	1
	INH, RIF, PZA, AMK, CM	1
	SM, INH, RIF, OFL, CM	1
6 drugs	SM, INH, RIF, EMB, PZA, ETH	2
	SM, INH, RIF, EMB, AMK, CM	2
	SM, INH, RIF, EMB, PZA, OFL	2
	SM, INH, RIF, EMB, ETH, OFL	1
	SM, INH, RIF, EMB, PZA, PAS	1
	INH, RIF, EMB, OFL, AMK, CM	1
7 drugs	SM, INH, RIF, EMB, ETH, AMK, CM	1
	SM, INH, RIF, EMB, OFL, AMK, CM	2
	SM, INH, RIF, EMB, PZA, ETH, OFL	1
	SM, INH, RIF, EMB, PZA, ETH, PAS	1
8 drugs	SM, INH, RIF, EMB, PZA, OFL, AMK, CM	6
	SM, INH, RIF, EMB, PZA, ETH, AMK, CM	2
9 drugs	SM, INH, RIF, EMB, PZA, ETH, OFL, AMK, CM	1

*Resistance profiles of XDR MTBC strains (n = 11). AMK, amikacin; CM, capreomycin; EMB, ethambutol; ETH, ethionamide; INH, isoniazid; KM, kanamycin; MDR, multidrug-resistant; MTBC, *Mycobacterium tuberculosis* complex; OFL, ofloxacin; PAS, para-aminosalicylic acid; PZA, pyrazinamide; RIF, rifampin; SM, streptomycin; XDR, extensively drug-resistant.

Appendix 1 Table 2. Likelihood scores for the top 10 substitution models for MDR TB in a longitudinal study, Serbia*

Substitution model	-lnL	AIC	∆ AIC (AIC ranking)	BIC	∆ BIC (BIC ranking)
All isolates					
**GTR**	**46521.4**	**93466.8**	**0.0 (1)**	**94904.4**	**0.0 (1)**
GTR+I	46521.5	93469.0	2.2 (2)	94913.4	9.0 (2)
GTR+G	46547.9	93521.8	55.0 (3)	94966.2	61.8 (5)
TPM1uf	46553.7	93525.4	58.7 (4)	94942.7	38.3 (3)
GTR+I+G	46548.7	93525.4	58.7 (5)	94976.7	72.3 (6)
TPM1uf+I	46554.2	93528.5	61.7 (6)	94952.6	48.1 (4)
TPM1uf+G	46577.8	93575.5	108.8 (7)	94999.6	95.2 (7)
TPM1uf+I+G	46581.1	93584.1	117.3 (8)	95015.0	110.6 (8)
HKY	46652.6	93721.2	254.5 (9)	95131.8	227.3 (9)
TrN	46652.1	93722.1	255.4 (10)	95139.5	235.0 (10)
TUR genotype					
TPM1uf	10101.0	20356.0	0.0 (1)	20878.2	0.0 (1)
TPM1uf+G	10100.4	20356.8	0.8 (2)	20885.8	7.6 (3)
**GTR**	**10098.6**	**20357.2**	**1.1 (3)**	**20899.7**	**21.5 (12)**
TPM1uf+I	10100.9	20357.8	1.7 (4)	20886.7	8.4 (4)
GTR+G	10098.0	20357.9	1.9 (5)	20907.2	29.0 (14)
GTR+I	10098.4	20358.9	2.8 (6)	20908.1	29.9 (15)
TPM1uf+I+G	10100.5	20359.0	3.0 (7)	20894.7	16.5 (8)
GTR+I+G	10098.0	20360.1	4.0 (8)	20916.1	37.9 (16)
**HKY**	**10106.7**	**20365.4**	**9.3 (9)**	**20880.8**	**2.5 (2)**
HKY+G	10106.1	20366.2	10.1 (10)	20888.4	10.2 (5)

*Models were calculated with jmodeltest 2.1 and statistical model selection based on Akaike and Bayesian information criteria. The best model is assumed to have the lowest criteria value. Boldface indicates substitution models used for Bayesian inference. AIC, Akaike information criteria; BIC, Bayesian information criteria;-lnL, log-likelihood value
Adapted from Posada D. jModelTest: phylogenetic model averaging. MolBiolEvol. 2008;25:1253–56.

Appendix Table 3. Path sampling results and model selection relative to the best substitution models in a longitudinal study of MDR TB, Serbia*

Dataset	Substitution model	Clock model	Demographic model	Marginal L estimate	∆ marginal L estimate	Substitution rate × 10^{-7} (SD)
GTR model selected as best substitution model						
TUR genotype	HKY	Strict (no tip dating)	Coalescent constant size	−10173.12	11.14	1.0 (fixed)
**TUR genotype**	**GTR**	**Strict (no tip dating)**	**Coalescent constant size**	**-10161.98**	**ref**	**1.0 (fixed)**
TUR genotype	HKY	Strict (tip dating)	Coalescent constant size	−10171.63	11.05	1.0 (fixed)
**TUR genotype**	**GTR**	**Strict (tip dating)**	**Coalescent constant size**	**-10160.58**	**ref**	**1.0 (fixed)**
TUR genotype	HKY	Relaxed, lognormal	Coalescent constant size	−10173·51	11.09	0.91 (0.62–1.21)
**TUR genotype**	**GTR**	**Relaxed, lognormal**	**Coalescent constant size**	**-10162·42**	**ref**	**0.92 (0.62–1.21)**
Strict clock with tip dating under coalescent constant population size selected as best demographic model						
TUR genotype	GTR	Strict (no tip dating)	Coalescent constant size	−10161.98	1.40	1.0 (fixed)
**TUR genotype**	**GTR**	**Strict (tip dating)**	**Coalescent constant size**	**-10160.58**	**ref**	**1.0 (fixed)**
TUR genotype	GTR	Relaxed, lognormal	Coalescent constant size	−10162.42	1.84	0.92 (0.62–1.21)
Likely city of infection inferred from a fast TB progression assumption selected for a discrete phylogeographical analysis of TUR genotype MDR strains						
**TUR genotype (city of infection–fast TB progression)**	**GTR**	**Strict (tip dating)**	**Coalescent constant size**	**-10219.54**	**ref**	**1.0 (fixed)**
TUR genotype (city of infection – slow TB progression)	GTR	Strict (tip dating)	Coalescent constant size	−10263.76	44.22	1.0 (fixed)

*Sampling results and model selection were determined from the change in marginal L estimates analyzing 50 path sampling steps and chain lengths of 10 million. Boldface indicates estimates relative to the best model. Adapted from Posada D. jModelTest: phylogenetic model averaging. MolBiolEvol 2008;25: 1253–56. TB, tuberculosis; MDR, multidrug-resistant.

Appendix Table 4. Comparison of demographic, clinical, epidemiologic, and laboratory characteristics of clustered patients with those of nonclustered patients in a longitudinal MDR TB study, Serbia

Characteristic	Whole-genome sequence cluster		p value
	Yes	No	
Sex			
M	52 (64.2%)	29 (35.8%)	0.226
F	11 (50.0%)	11 (50.0%)	
Age group			
15–24	7 (87.5%)	1 (12.5%)	0.603
25–34	2 (28.6%)	5 (71.4%)	
35–44	16 (72.7%)	6 (27.3%)	
45–54	13 (50.0%)	13 (50.0%)	
55–64	18 (66.7%)	9 (33.3%)	
65+	7 (53.8%)	6 (46.2%)	
Refugee status			
Yes	4 (100.0%)	0 (0.0%)	0.155
No	59 (59.6%)	40 (40.4%)	
Region			
Belgrade	19 (65.5%)	10 (34.5%)	0.280
Vojvodina	8 (44.4%)	10 (55.6%)	
Central Serbia	34 (64.2%)	19 (35.8%)	
Bosnia and Herzegovina	2	1	
Previous tuberculosis treatment			
Yes	32 (57.1%)	24 (42.9%)	0.361
No	31 (66.0%)	16 (34.0%)	
Alcoholism			
Yes	6 (42.9%)	8 (57.1%)	0.131
No	57 (64.0%)	32 (36.0%)	
Diabetes mellitus			
Yes	3 (37.5%)	5 (62.5%)	0.256
No	60 (63.2%)	35 (36.8%)	
Schizophrenia			
Yes	23 (92.0%)	2 (8.0%)	<0.001
No	40 (51.3%)	38 (48.7%)	
Cardiovascular disease			
Yes	4 (80.0%)	1 (20.0%)	0.646
No	59 (60.2%)	39 (39.8%)	
Chronic obstructive respiratory disease			
Yes	6 (60.0%)	4 (40.0%)	1.000
No	57 (61.3%)	36 (38.7%)	
Microscopy			
Positive	42 (55.3%)	34 (44.7%)	0.051
Negative	20 (76.9%)	6 (23.1%)	
Unknown	1		
Hospitalization			
Bela Crkva*†			
Yes	26 (92.9%)	2 (7.1%)	<0.001
No	30 (41.7%)	42 (58.3%)	
Ozren*‡			
Yes	30 (45.5%)	36 (54.5%)	<0.001
No	28 (82.4%)	6 (17.6%)	
Unknown	4	1	
Cavern presence			
Yes	41 (56.9%)	31 (43.1%)	0.347
No	15 (68.2%)	7 (31.8%)	
Unknown	7	2	

*Two patients with clustered MDR-MTBC isolates were hospitalized both in Bela Crkva and Ozren.
†Hospital for treatment of all psychiatric patients with concomitant respiratory illnesses in Serbia;
‡Hospital for the initial treatment phase of all patients diagnosed with tuberculosis and multidrug-resistant tuberculosis in Serbia.

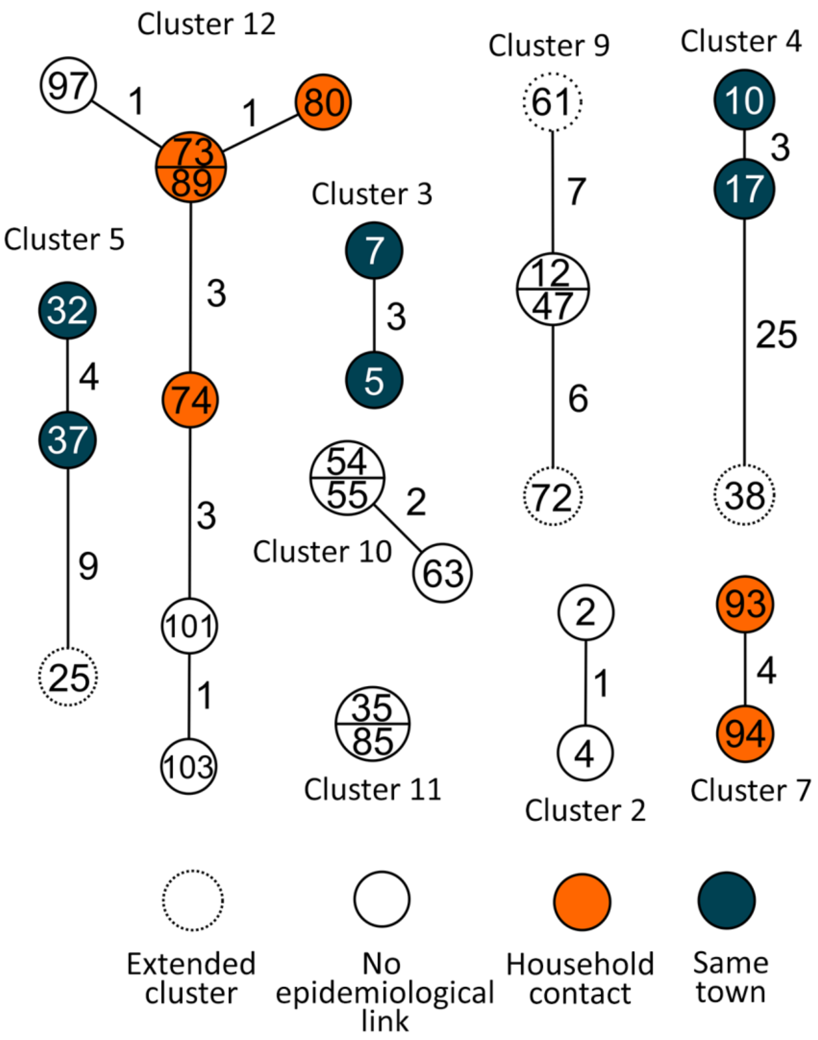

Appendix 1 Figure 1. Household and social links within WGS predicted molecular clusters. Genetic distances are based on maximum parsimony analysis. Each circle represents a node of patients who were infected with genetically identical isolates. SNP distances are annotated on branches. Known epidemiologic links between patients are color coded. WGS, whole genome sequencing; SNP, single nucleotide polymorphism.

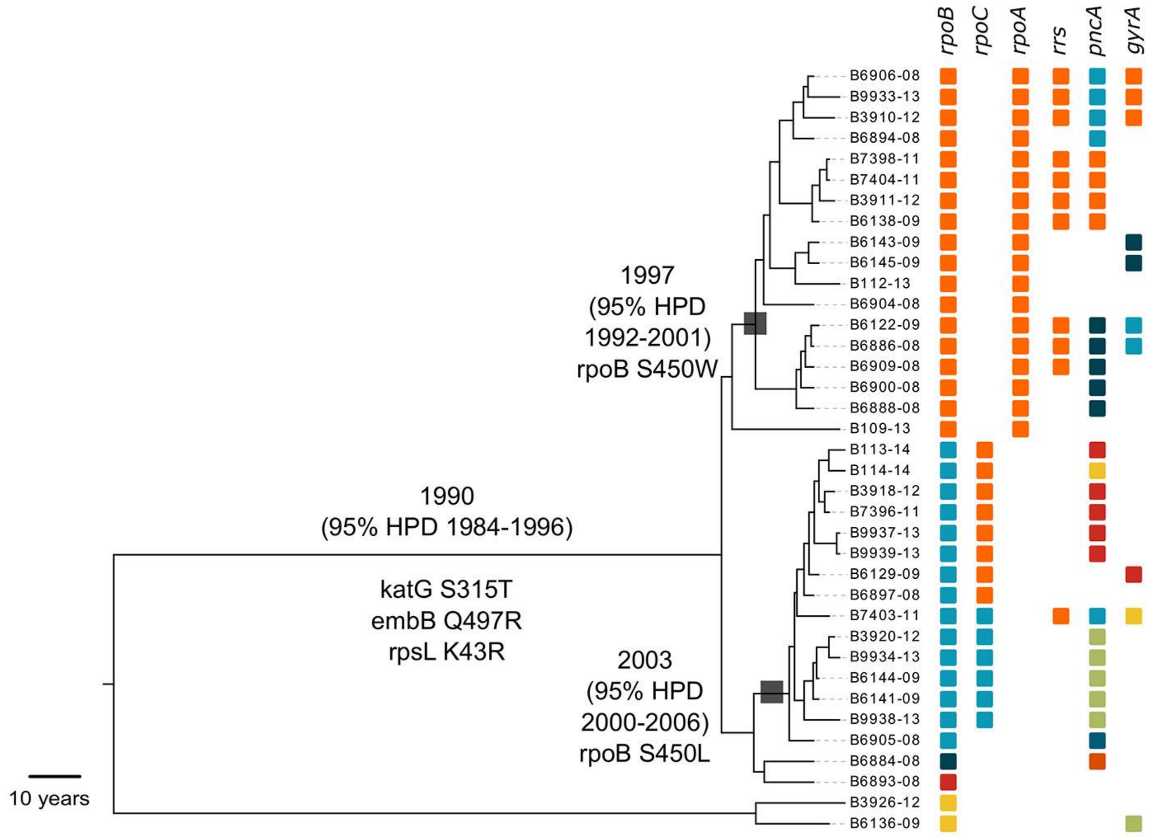

Appendix 1 Figure 2. Temporal acquisition of resistance to rifampin (mediated by *rpoB* mutations) among 37 lineage 4.2.2.1 (TUR-genotype) MDR MTBC isolates from Serbia. Time-scaled phylogeny (maximum clade credibility tree) derived from a Bayesian coalescent constant size model of 37 lineage 4.2.2.1 (TUR-genotype) MDR MTBC isolates. The analysis used a GTR substitution model, a strict molecular clock fixed to 1×10^{-7} substitutions per site per year, and tip dating (best model according to path sampling analysis, Appendix 1 Table 3). Rifampicin resistance was acquired at least twice. Within the Serbian TUR-outbreak strain the upper clade shares *rpoB* S450W (orange), the lower clade shares *rpoB* S450L. Most recent common ancestors of both clades (with a minimum posterior node support of 0.5) were dated to 1998 (95% HPD 1993–2001) and 2003 (95% HPD 2000–2005). Values are rounded to full years. Putative compensatory mutations (in *rpoC* and *rpoA*) and other resistance mediating mutations identified among TUR-strains are color coded. Internal node support, i.e. posterior density, for all strains (including B109–13) with *rpoB* S450W, *rpoA* P25R (both in orange) is 0.37. GTR, general time reversible; HPD, highest posterior density; MDR, multidrug-resistant; MTBC, *Mycobacterium tuberculosis* complex.

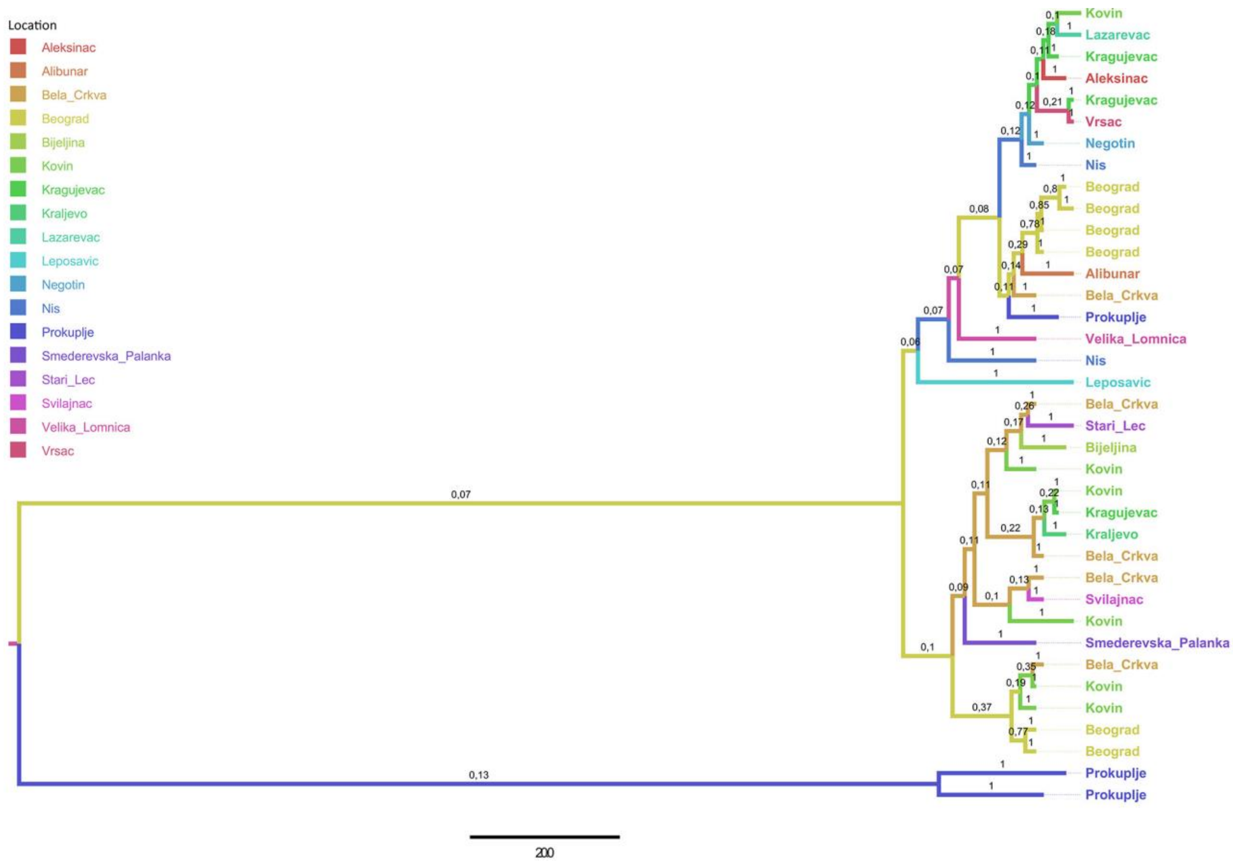

Appendix 1 Figure 3. Hypothetical origin of MTBC TUR-genotype outbreak strains assuming a slow TB progression of infected patients. Location annotated time-scaled phylogeny (maximum clade credibility tree) is derived from a Bayesian discrete trait phylogeographical analysis of 37 lineage 4.2.2.1 (TUR-genotype) MDR MTBC isolates. Branches are color coded by the most likely place of infection, assuming a slow TB progression hypothesis (patients diagnosed in the hospital for treatment of psychiatric inpatients with concomitant respiratory illnesses, Bela Crkva Hospital, within the first 2 years after admission were assumed to be infected in the initial hospital or their hometown, and administered as latent TB case). Branches are annotated with probable locations. The origin of the TUR-outbreak strain, applying a slow disease progression hypothesis, is ambiguous, with 7% location support for the most recent common ancestor originating from Belgrade (other location probabilities rank lower). MDR, multidrug-resistant; MTBC, *Mycobacterium tuberculosis* complex; TB, tuberculosis.

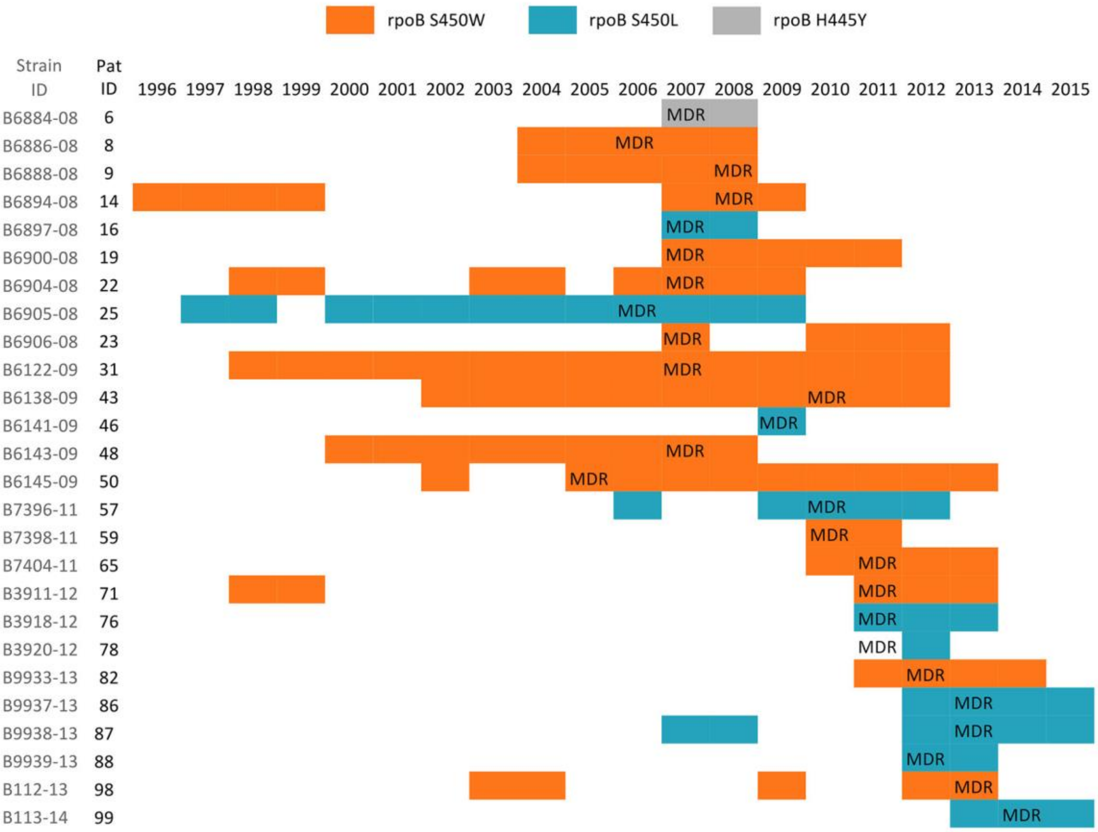

Appendix 1 Figure 4. Coexistence of 2 MDR MTBC transmission chains in the psychiatric hospital Bela Crkva, Eastern Serbia. The figure shows all (n = 26) Bela Crkva Hospital patients infected with lineage 4.2.2/TUR genotype MDR MTBC strains. Bars represent hospitalization time; MDR denotes the year of MDR TB diagnosis. The 2 MTBC strain types shown in orange (*rpoB* S450W) and blue (*rpoB* S450L) carry individual rifampin resistance–mediating mutations. Gray indicates *rpoB* H445Y. ID, identity number; MDR, multidrug-resistant; MTBC, *Mycobacterium tuberculosis* complex; Pat, patient; TB, tuberculosis.
